# Optimization and Validation of Thermal Desorption Gas Chromatography-Mass Spectrometry for the Determination of Polycyclic Aromatic Hydrocarbons in Ambient Air

**DOI:** 10.1155/2018/8734013

**Published:** 2018-05-02

**Authors:** Iñaki Elorduy, Nieves Durana, José Antonio García, María Carmen Gómez, Lucio Alonso

**Affiliations:** Chemical and Environmental Engineering Department, School of Engineering, University of the Basque Country, Alameda de Urquijo s/n, 48013 Bilbao, Spain

## Abstract

Thermal desorption (TD) coupled with gas chromatography/mass spectrometry (TD-GC/MS) is a simple alternative that overcomes the main drawbacks of the solvent extraction-based method: long extraction times, high sample manipulation, and large amounts of solvent waste. This work describes the optimization of TD-GC/MS for the measurement of airborne polycyclic aromatic hydrocarbons (PAHs) in particulate phase. The performance of the method was tested by Standard Reference Material (SRM) 1649b urban dust and compared with the conventional method (Soxhlet extraction-GC/MS), showing a better recovery (mean of 97%), precision (mean of 12%), and accuracy (±25%) for the determination of 14 EPA PAHs. Furthermore, other 15 nonpriority PAHs were identified and quantified using their relative response factors (RRFs). Finally, the proposed method was successfully applied for the quantification of PAHs in real 8 h-samples (PM_10_), demonstrating its capability for determination of these compounds in short-term monitoring.

## 1. Introduction

Polycyclic aromatic hydrocarbons (PAHs) are a class of persistent organic pollutants (POPs) comprising hundreds of individual substances. These compounds contain two or more fused aromatic rings (made up of carbon and hydrogen atoms) in linear, angular, or cluster arrangements [1]. They are semivolatile organic compounds (SVOC); thus, they are present in the atmosphere in both the gas and the particulate phases as well as dissolved or suspended in precipitation (fog or rain) [2].

PAHs are as by-products of incomplete combustion processes of organic matter [3], and primarily emitted from anthropogenic sources [4], being the mobile and major sources in urban areas [5, 6]. Their harmful health effects and persistence pose an environmental concern. Thus, these compounds were among the first atmospheric pollutants identified as suspected carcinogens [7]. Moreover, PAHs belong to the group of POPs included in the list of 16 POPs specified by the UNECE Convention on Long-range Transboundary Air Pollution Protocol on Persistent Organic Pollutants [8, 9]. Due to these features, the United States Environmental Protection Agency (US-EPA) has listed 16 of them as priority pollutants (16 EPA PAHs) [10]. The most toxic PAHs (5 and 6 rings) are linked to the particulate matter [11, 12]. Accordingly, many air pollution studies have been focused on PAHs bound to particulate matter, particularly PM_10_ and PM_2.5_ in order to assess their concentration, distribution, and sources.

Air monitoring for PAHs in urban areas is an important issue because the risk associated with human exposure is higher considering the population density [13, 14]. However, PAH data in urban air show large spatial and temporal uncertainties because of the complex sampling and analytical procedures required.

Sampling of particulate PAHs is mostly done by the collection of them on a filter (quartz or glass fiber), using high- or low-volume samplers [15–17]. Once the PAHs have been collected, they have to be extracted for the final determination. The extraction of PAHs from multiple matrices is a difficult step. PAHs are found in the environment in very low concentrations; consequently, an effective extraction method, able to quantitatively separate the analytes from the matrix, is required. The widely used method is solvent desorption of sampling media (Soxhlet extraction, accelerated solvent extraction, microwave-assisted extraction, and ultrasonic-assisted extraction) followed by analysis of the compounds of interest by GC-MS (gas chromatography coupled to mass chromatography) or high-performance liquid chromatography coupled with florescence detection (HPLC-FLD) [18, 19], where the detection methods allow cutting most of analytical interferences.

The use of toxic organic solvents in the solvent-based extraction methods causes added difficulties with sample handling and generates large amounts of solvent waste, which is costly and can generate additional environmental problems. Furthermore, the sensitivity of the current analytical procedures limits time resolution of measurements; thus, most of the urban pollution studies rarely achieved temporal resolution measurements better than 24 h. Since the PAH composition of aerosols can vary according to the diurnal changes in the sources, meteorological conditions and atmospheric reactivity [20], the time resolution of 24 h seems not sufficient to comprehend their variability, fate, and behavior in the atmosphere [21]. For these reasons, the development of simpler and sensitive methods or the improvement of the existing ones is of great interest, for the detection, determination, and monitoring of PAHs.

In recent years, alternative analytical procedures for PAHs based on the use of solvent-free extraction methods have been studied [22, 23]. Thermal desorption (TD) involves heating sample materials or sorbents in a flow of inert carrier gas, so that retained organic volatiles and semivolatiles are released and transferred or injected into the analytical system (e.g., into the carrier gas stream of the GC column).

The power and potential of TD allow configuring the technique in multiple adsorption-desorption stages, thus enhancing the concentration of the compounds of interest and detection limits. This higher sensitivity may provide shorter sampling times or lower sampling volumes. Another benefit of TD is that it is often possible to quantitatively retain target compounds during one or more of the trapping stages, while unwanted, for example, water and/or permanent gases, is selectively purged to vent. This avoids the entrance of unwanted compounds into the analytical system that could generate interferences during the analysis and/or damage to the equipment.

This work has tested and optimized different TD-GC/MS operation conditions in order to develop the best method that is able to sample and analyze airborne PAHs in particulate phase. The TD-GC/MS method was later validated by using a Standard Reference Material (SRM) 1649b urban dust and comparing with the conventional method based on solvent extraction (Soxhlet extraction-GC/MS). Moreover, the method was applied to measure PAH levels of 8 h PM_10_ samples in ambient air.

## 2. Materials and Methods

### 2.1. Reagents and Materials

A liquid certificated mixture of 16 EPA PAHs (2000 *μ*g·mL^−1^, SV Calibration Mix 5, Restek Corporation, USA) and a liquid deuterated mixture (200 *μ*g·mL^−1^, predeuterated internal standard PAH Mixture 6, Chiron AS, Norway) were used during the study. In Soxhlet extraction, decafluorobiphenyl, 4,4′-dibromooctafluorobiphenyl, 4,4′-dibromobiphenyl. (Restek, 2000 *μ*g·mL^−1^), and indeno [1,2,3-cd]pyrene-d12 (Chiron, 100 *μ*g·mL^−1^) were used as recovery standard for the assessment of extraction efficiency. Solutions were prepared by appropriate dilution in methanol, HPLC grade (99.9%, Lab-Scan Analytical Sciences, Poland).

The method was validated using the Standard Reference Material (SRM) 1649b urban dust, obtained from the National Institute of Standards and Technology (NIST, Gaithersburg, MD, USA).

### 2.2. TD-GC/MS Method

Sampling tubes (stainless steel tube of 5 mm outer diameter × 90 mm length) packed with filter were analyzed by using TD-GC/MS. The 16 EPA PAHs and deuterated PAHs were spiked on two one-eighth parts of a 47 mm quartz fiber filter (Whatman International Ltd., UK). These portions, suitably folded, were introduced into the sampling tubes.

Prior to use, the packed sampling tubes were conditioned by thermal cleaning under a helium flow rate of 100 mL·min^−1^ at 350°C for 30 min.

The NIST Standard Reference Material 1649b was handled in a similar way. Samples of the urban dust (10 mg) were weighed and placed on a one-eighth section of a 47 mm quartz fiber filter which was rolled and put into the sampling tube. Silanized glass wool (Supelco Inc., Bellefonte, USA) was introduced at the end and at the head of the desorption tubes in order to prevent system contamination.

Prior to use, filters and glass wool plugs were heated in a muffle furnace at 500°C for 24 h to remove trace organic compounds.

PAHs analysis was carried out using an automatic thermal desorber unit (Turbomatrix 150 ATD, Perkin Elmer S.L., USA) coupled by a fused silica capillary transfer line (5 m length × 0.32 mm I.D.) to a GC/MS detector (Clarus 500, Perkin Elmer S.L., USA). The chromatographic separation of PAHs was conducted on a Meta. X5 (silphenylene phase) capillary column: 30 m length × 0.25 mm I.D. × 0.25 mm film thickness (Teknokroma, Spain).

The helium gas carrier pressure employed in the GC/MS system was 145 kPa, and the column temperature was programmed as follows: initial temperature 100°C for 3 min, ramp of 10°C·min^−1^ until 250°C, ramp of 5°C·min^−1^ until 320°C, and finally temperature held at 320°C for 10 min. The total analysis time was 42 min per sample. The temperature of both the transfer lines (from TD to GC and from GC to MS) was held at 280°C, whereas the source temperature was 250°C. Simultaneous full scan (SCAN) and selective ion monitoring (SIM) modes were used for the identification and quantification of PAHs.  Table 1 shows, according to their elution order, the PAHs determined in this study with their quantification ions. Supplementry Figures S1–S3 show the representative SCAN chromatograms of the 16 EPA and deuterated PAHs.

### 2.3. Soxhlet Extraction-GC/MS Method

Between 300 and 500 mg of the NIST SRM 1649b urban dust was weighted and placed on one-eighth of a 150 mm prebaked (at 500°C for 24 h) quartz fiber filter (Whatman International Ltd.). Before folding the filter, it was spiked with the recovery standards.

Soxhlet extraction was performed by using Büchi extraction system B-811 (BÜCHI, Switzerland), an automated system that can be used to perform extraction according to the original Soxhlet principle. The samples were extracted with hexane using the Soxhlet Warm mode. This mode increases the solubility of the analytes, allowing an optimal extraction in 3 hours [24].

After the extraction process, the extracts of 5 mL were concentrated by a stream of dry nitrogen to a volume less than 0.5 mL. Finally, these extracts were diluted to 1.5 mL with methanol and spiked with deuterated PAHs.

Two-microliters  of aliquots from each extract was injected into the GC/MS with split mode.  Table 2 collects the timed events and the oven program used in the GC/MS during the validation of the Soxhlet method.

Also, in this method, the GC/MS used simultaneously the SCAN and SIM mode for the identification and quantification of PAHs.

### 2.4. Sample Collection

Airborne particulate matter (PM_10_) samples were collected on preheated (at 500°C for 24 h) quartz fiber filters (150 mm diameter, Whatman International Ltd., United Kingdom) using a high-volume sampler (Digitel DHA-80, Digitel Elektronik AG, Switzerland) with a flow rate of 30 m^3^·h^−1^. DHA-80 stores 15 filters stretched in filter holders that are changed automatically at the preset time. DHA-80 has integrated temperature control in the filter storage section; in this way, the used filters can be stored at low temperatures (in this study at 4°C) after sampling.

Collected filters were put into individual Petri dishes, wrapped in aluminum foil, and kept in a 4°C freezer until analysis (<15 days) according to ISO 12884:2000 [25].

## 3. Results and Discussion

### 3.1. Optimization of Thermal Desorption Method

The thermal desorption process can be divided into two main stages: tube and trap desorption. In the first stage, target compounds are thermally desorbed from the sampling tube and transferred to the cold trap, where they are concentrated. After completing the primary desorption, the trapped compounds are released by quick heating of the trap and swept through the heated transfer line to the GC column.

To obtain the best analytical conditions in terms of sensitivity and reproducibility, different parameters in each desorption stage were tested.

For these tests, 1 *μ*L of the 16 EPA PAHs solutions of 20 ng·*μ*L^−1^ were spiked in sampling tubes packed with portions of quartz fiber.

#### 3.1.1. Primary Desorption (Tube Desorption)

The conditions in the tube oven during this stage are key to guarantee an efficient desorption; thus, parameters such as the temperature, time, and flow in the tube oven were studied to optimize this process.

**Figure 1:**
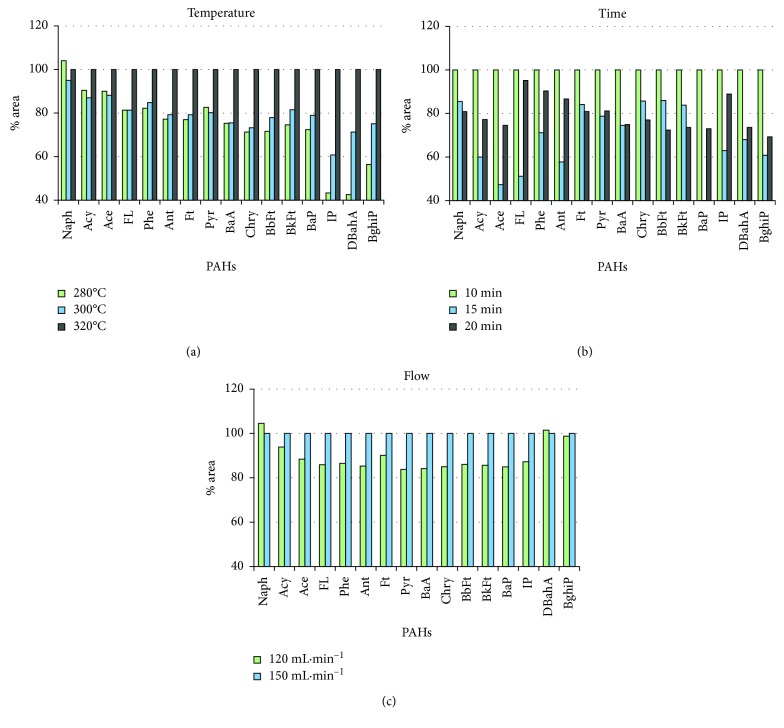
Area (in %) for each of the 16 EPA PAH obtained in the study of primary desorption conditions (desorption temperature, time, and flow) for sampling tubes (*n*=5) packed with filter. % Areas at 280°C and 300°C are compared to % areas at 320°C chosen as 100% (a). % Areas at 15 min and 20 min are compared to % areas at 10 min chosen as 100% (b). % Areas at 120 mL·min^−1^ is compared to % areas at 150 mL·min^−1^ chosen as 100% (c).

Different values of desorption temperatures, times, and flows were tested, considering factors such as the packing/sample matrix stability, the lability of the components of interest, and the temperature limitations of the system.  Figure 1 shows the area of chromatographic peak for each of the 16 EPA PAHs (in %) obtained for each test.

The results demonstrated that an increase in the temperature of the oven tube enhances the desorption of particulate PAHs (Figure 1(a)). This improvement was remarkable for high molecular weight PAHs (IP, DBahA, and BghiP). A value of 320°C was selected as temperature in the first desorption stage. Regarding the time, the lowest value in the test (10 min) clearly showed significantly higher areas for most compounds (Figure 1(b)), indicating a more efficient desorption. This value was selected as desorption time in the tube. Finally, higher desorption flows enable better desorption of PAHs (Figure 1(c)). Flows higher than 150 mL·min^−1^ are not recommended, as they can generate problems in maintaining low temperatures in the trap zone during the first desorption stage [26]. Therefore, a flow of 150 mL·min^−1^ was selected as the optimal value.

#### 3.1.2. Secondary Desorption (Trap Desorption)

To enhance PAH desorption from the trap (a quartz tube packed with glass wool), its high temperature has to be as high as possible. This temperature depends on the trap packing and equipment stability as well as on the target compounds. In this study, a value of 320°C (the value recommended by the manufacturer) was set, while its low temperature (values of −15, −10, and −5°C) and desorption time (values of 4, 6, and 12 min) were tested.

**Figure 2:**
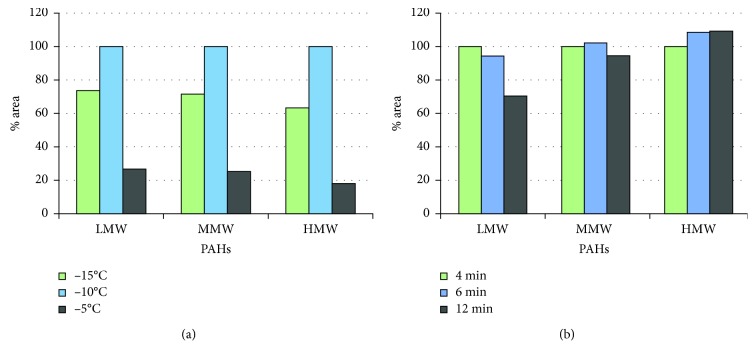
Area (in %) for LMW (low molecular weight), MMW (middle molecular weight), and HMW (high molecular weight) PAHs obtained in the study of the low trap temperature (a) and of the trap time (b) for sampling tubes packed with filter (*n*=5). % Areas at −15°C and −5°C are compared to % areas at −10°C chosen as 100% (a). % Areas at 6 min and 12 min are compared to % areas at 10 min chosen as 100% (b).

The area of chromatographic peak (in %) for low (2-3 rings: Naph, Ace, Acy, FL, Phe, and Ant), middle (4 rings: Ft, Pyr, BaA, and Chry), and high (5-6 rings: BbFt, BkFt, BaP, IP, DBahA, and BghiP) molecular weight PAHs obtained for each test are shown in Figure 2.

The temperature in the Peltier trap is a critical parameter in secondary desorption (Figure 2(a)), showing significant changes in the sensitivity for different values. The temperature of −10°C revealed the best results.

In the study of the trap desorption time (Figure 2(b)), the results demonstrated that longer values do not implicate a higher efficiency and consequently a better detection, 6 min showed a better response than 12. This is especially significant with the lightest PAHs (LMW) which could be affected by the exposure to high temperatures, generating losses. By contrast, the heavier PAHs (MMW and HMW) showed higher concentrations after longer trap desorption times because they could need more time to be completely desorbed. Due to this, a trap duration of 6 min was selected as this value presented good desorption for 16 target PAHs.

#### 3.1.3. Inlet and Outlet Split Flows

In order to enhance the process of two-stage thermal desorption, a double split mode was used. Therefore, the inlet (split flow as the tube is desorbed) and outlet (split flow as the trap is desorbed) split flows were also tested.

The inlet split flow plays an important role during primary desorption. This maintains a relatively high carrier gas flow through the sample tube, while at the same time establishes a reasonably low flow through the cold trap, aiding the complete removal from the sample tube and analyte retention. The deactivation of the inlet split (0 mL·min^−1^) generated a significant improvement in PAH desorption because the complete sample, without purge, arrived at the cold trap. With the increase of inlet split, the sensitivity decreased. Although in this study, an inlet split flow of 0 mL·min^−1^ showed the best results; it is recommended to activate this split in order to avoid the presence of unwanted compounds (permanent gases and water) in the trap. These could reduce the trap lifetime and interfere in the analysis. In order to find a compromise between these rules, an intermediate flow (23 mL·min^−1^) was considered as the optimal value.

The outlet split flow also plays an important function in the trap desorption: (1) adapting the effluent flow to a capillary column flow, it avoids the system saturation and (2) facilitating the release of the analytes, it guarantees a high enough flow through the trap during desorption. According to the manufacturer, at least 10 mL·min^−1^ of outlet split is necessary to minimize the air/water background on a mass spectrometer when atmospheric samples are analysed [26]. In this study, the results obtained for outlet split flows demonstrated that the increase of this parameter reduces the sensitivity of the technique, with losses becoming significant between 10 and 20 mL·min^−1^. Therefore, the manufacturer's recommended flow (10 mL·min^−1^) was selected as the optimal value.

Finally, Table 3 summaries the optimized values for thermal desorption.

### 3.2. Desorption Efficiency

Once optimized, the efficiency of two-stage thermal desorption was studied. The efficiency was calculated by the following expression:

(1) $$\begin{array}{c} E\left(\%\right)=\left(\frac{A}{A+A^{*}}\right)\times 100, \end{array}$$

where *E* is the efficiency in %, *A* is the peak area of the analyte obtained from desorption of the sampling tube (previously loaded with PAHs), and *A* ^∗^ is the peak area of the analyte obtained when the same sampling tube or the trap was desorbed the second time.

**Figure 3:**
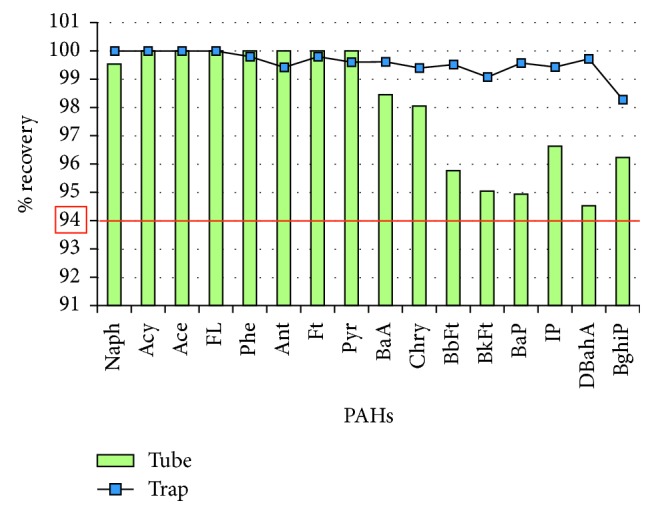
Recovery (in %) of the 16 EPA PAHs (particulate phase) in each stage of the thermal desorption.

Figure 3 shows the tube and trap efficiencies obtained for each particulate PAH. The technique demonstrated a good efficiency with recoveries of the PAHs in the tube and trap higher than 94%.

### 3.3. TD-GC/MS Validation and Comparison with Soxhlet-GC/MS

In order to determine the performance of the method when applied to atmospheric PM samples, this was tested using the Standard Reference Material (SRM) 1649b urban dust.

The accuracy, repeatability, and recovery of the method were calculated by adding known amounts (approximately 10 mg) of the SRM 1649b to a one-eighth section of blank filters (*n*=10). Before the analysis, filters were spiked with 1 *μ*L of the deuterated PAH internal standard solution (25 ng·*μ*L^−1^).

Table 4 shows the results obtained for each PAH by using the TD-GC/MS method, comparing the calculated concentration with the certified values.

Although the column used in this study demonstrated a good resolution for the 16 EPA PAHs, the presence of other PAHs in the urban dust can generate coelution problems with the target compounds [27]. Some PAH pairs such as BbFt and DBahA coeluted with the benzo[j]fluoranthene (BjFt) and dibenzo[a,c]anthracene (DBacA), respectively.

The TD-GC/MS method showed good precision with mean RSD values of 12.2. The accuracy of the TD-GC/MS method ranged from −22.8% to 25.1%, while the average recovery efficiency was 96.7. These performance parameters of the TD-GC/MS method accomplish the quality objectives for ambient air PAHs stated by ISO 12884:2000 [25], which establishes a precision of ±25%, an accuracy of ±20%, and a recovery efficiency between 75 and 125%. These requirements are accomplished for most PAH; however, there are some exceptions. The lowest molecular weight PAHs (Naph and Acy), with excessively high recoveries, confirmed the overestimation of these compounds when analyzed by using the TD-GC/MS method. These compounds could suffer losses during the sample preparation due to their high volatility. Besides, the presence of interfering compounds in the SRM and the low concentration of Acy could explain these overestimations. Therefore, this method was not applicable to the Naph and Acy analysis.

In order to demonstrate the efficiency of the TD method as compared with other analytical methods, the conventional method (Soxhlet extraction-GC/MS) was also tested (Table 5). Between 300 and 500 mg of the NIST SRM 1649b urban dust was placed on one-eighth of a 150 mm prebaked quartz fiber filter, which was spiked with 1 *μ*L of a solution (0.5 ng·*μ*L^−1^) of the recovery standards. After the extraction process, the obtained extracts were spiked with 25 *μ*L of a deuterated PAH solution (20 ng·*μ*L^−1^).

The results of the Soxhlet extraction-GC/MS method showed a good recovery for 4-, 5-, and 6-ring PAHs with values between 72.8 and 131%; whereas the lightest PAHs (2- and 3-ring PAHs), except Phe, showed low recovery (<70%). The loss of these analytes during the extraction process in the Soxhlet Warm mode could be the main reason for these low recoveries. In the case of DBahA, although its coelution with DBacA was considered, its recovery continued to be high (>200%). This indicates an overestimation in the determination of this compound by the Soxhlet process. Regarding precision and accuracy, the Soxhlet extraction-GC/MS showed worse results, with an average precision of 34.9 and values of accuracy out of the limits ±20% for some PAHs.

Comparing both methods, the TD-CG/MS method demonstrated a better performance (good recovery, precision, and accuracy) for the determination of PAHs (except for Naph and Acy). By contrast, the manipulation of the samples in the Soxhlet process meant losses of the light PAHs (2- and 3-ring) and an overestimation of some PAHs, especially of the DBahA.

Regarding the detection limits, the average instrument detection limit (IDL) of the TD-GC/MS method was 0.04 ng and the average method detection limit (MDL), assuming a total sample volume 240 m^3^ (30 m^3^·h^−1^ for 8 h), was 2.89 × 10^−3^ ng·m^−3^ [28].

### 3.4. Extension of the Scope to Other PAHs

Although most environmental studies are focused on the analysis of 16 PAH listed by US-EPA, it could be interesting to determine other PAHs in order to have a better characterization of these compounds in terms of toxicity and sources. For this reason, besides the 16 EPA PAHs, other 15 PAHs were determinated by using TD-GC/MS.  Table 1 shows, according to their elution order, the 16 EPA PAHs, the deuterated PAHs, and the 15 nonpriority PAHs.

Because SRM 1649b contains other compounds besides the 16 EPA PAHs, it was used to identify 15 nonpriority PAHs and to quantify them by relative response factors (RRFs). Supplementry Figures S4–S7 show the SIM chromatograms of the target PAHs (16 EPA PAHs + 15 PAHs) obtained in the analysis of SRM 1649b. For quantification, the RRFs for each nonpriority PAH were calculated by the following equation:

(2) $$\begin{array}{c} \mathrm{RR}F_{\mathrm{PAH}}=\frac{A_{\mathrm{PAH}}\cdot C_{\mathrm{ref}\cdot \mathrm{PAH}}}{A_{\mathrm{ref}\cdot \mathrm{PAH}}\cdot C_{\mathrm{PAH}}}, \end{array}$$

where *A*_PAH_ is the peak area of nonpriority PAH, *A*_ref·PAH_ is the peak area of reference PAH compound, C_PAH_ is the nonpriority PAH concentration in the NIST dust, and *C*_ref·PAH_ is the reference PAH concentration in the NIST dust.

Reference PAHs were selected according to the following criterion: the nearest of the 16 EPA PAHs to each new one, which provides the least variation in the RRF.  Table 6 collects the reference PAHs, RRFs, and the relative standard deviations (RSDs) for each nonpriority PAH.

The nonpriority PAHs showed a range of RRFs between 0.31 and 4.74, with RSD of less than 15% for most compounds. In the case of Ret, the low chemical similarity between this compound and its reference PAH (BaA) could explain the poor precision in the determination of its RRF (>20%).

### 3.5. Application to Real Samples

After validation, the method described in this study was applied to extract and analyze samples collected in the city of Bilbao, Spain (longitude 2°56′56.24″W, latitude 3°15′44.86″N). Bilbao city is the most populated area in the Basque Country and the tenth largest in Spain (approximately 350,000 in the city and 1 million inhabitants in the metropolitan area). In this urban area, local traffic and stationary emissions from the surrounding industries are considered as the major sources of atmospheric pollutants [28].

During seven consecutive days per month, eight-hour PM_10_ samples were collected at a flow rate of 30 m^3^·h^−1^.

A total of 182 PM_10_ samples were collected over 9 months (between July 2013 and June 2014). Each sample was randomly cut into 8 portions of 1 cm^2^ and introduced into the sampling tube and analyzed using the optimized method. This was performed in the same way as other studies [29, 30], which demonstrated good homogeneity results when using small sections of the filters.

Table 7 shows the descriptive statistics (number of valid data, mean, standard deviation, minimum, maximum, 5th, and 95th percentiles) for individual PAH concentrations measured by using the TD-GC/MS method in the city of Bilbao (urban area). PAHs which showed overestimation in the SRM analysis (Naph and Acy) or poor precision in the RRF determination (Ret) were not measured in the real samples.

The average concentration of individual EPA PAHs in Bilbao ranged from 0.04 ± 0.05 to 0.50 ± 0.76 ng·m^−3^, whereas the nonpriority PAHs were between 0.01 ± 0.01 and 0.26 ± 0.32 ng·m^−3^. The EPA PAHs reported minimum values between 4.00 × 10^−3^ and 0.03 ng·m^−3^ for most of the compounds, which are between 1.1 and 14.6 times the MDL, showing the suitability of the proposed method to determine particle-bound PAHs in real PM_10_ samples. Although the minimums of IP and DBahA were below their MDL, these values meant only the 5% or less of the measured samples.

Among compounds, BbFt was the major contributor to total PAHs (average concentration of 0.5 ± 0.76 ng·m^−3^), followed by Pyr (0.27 ± 0.23 ng·m^−3^), Ft (0.26 ± 0.22 ng·m^−3^), BeP (0.26 ± 0.32 ng·m^−3^), and Chry (0.22 ± 0.24 ng·m^−3^). The high presence of these compounds in PM_10_ fraction has been reported by previous studies [31, 32] in urban areas with traffic loads.

## 4. Conclusions

The method developed in this study, based on thermal desorption, showed a good efficiency for the determination of particle-bound PAHs. The use of a solvent-free extraction technique has showed numerous advantages (less sample manipulation and analysis time, reduced exposure risk, and higher sensitivity and reliability) that enable a better performance (good recovery, precision, and accuracy) for the determination of particle-bound PAHs; however, the lowest molecular weight PAHs (Naph and Acy) could be overestimated by this methodology.

Parameters such as tube and trap temperature, time, desorption, and split flows (inlet and outlet) were critical in the thermal desorption of PAHs. The optimized TD-GC/MS method showed an efficient desorption of PAHs with recoveries higher than 94%.

The results obtained in the validation of TD-GC/MS by standard reference material (urban dust) demonstrated that this is a reliable method to determine particulate PAHs in aerosol samples (good precision and accuracy), with average recovery efficiency of 96.67 and a mean RSD value of 12.18. Comparing with the conventional method Soxhlet-GC/MS, the TD-CG/MS method demonstrated a better performance for the determination of PAHs. Besides 16 EPA PAHs, the TD-GC/MS method demonstrated its ability to quantify other PAHs in aerosol samples.

Finally, the method was successfully applied for the quantification of PAHs in real PM_10_ samples collected with a time resolution of 8 h.

## Figures and Tables

**Table 1:** Abbreviations and quantification ions of PAHs determined by the TD-GC/MS method.

PAH	Abbreviation	Ion (m/z)
Naphthalene-d_8_^b^	Naph-d_8_	136
Naphthalene^a^	Naph	128
Biphenyl-d_10_^b^	Bph-d_10_	164
Acenaphthylene^a^	Acy	152
Acenaphthene^a^	Ace	154
Fluorene^a^	FL	166
Phenanthrene-d_10_^b^	Phe-d_10_	188
Phenanthrene^a^	Phe	178
Anthracene^a^	Ant	178
Fluoranthene^a^	Ft	202
Pyrene-d_10_^b^	Pyr-d_10_	212
Pyrene^a^	Pyr	202
Benzo[ghi]fluoranthene^c^	BghiFt	226
Benzo[c]phenanthrene^c^	BcP	228
Cyclopenta[cd]pyrene^c^	CPP	226
Benzo[a]anthracene-d_12_^b^	BaA-d_12_	240
Benzo[a]anthracene^a^	BaA	228
Triphenylene^c^	Triph	228
Chrysene^a^	Chry	228
Retene^c^	Ret	234
Benzo[b]fluoranthene^a^	BbFt	252
Benzo[j]fluoranthene^c^	BjFt	252
Benzo[k]fluoranthene^a^	BkFt	252
Benzo[a]fluoranthene^c^	BaFt	252
Benzo[e]pyrene^c^	BeP	252
Benzo[a]pyrene-d_10_^b^	BaP-d_10_	264
Benzo[a]pyrene^a^	BaP	252
Perylene^c^	Per	252
Dibenzo[a,j]anthracene^c^	DBajA	278
Indeno[1,2,3-cd]pyrene^a^	IP	276
Dibenzo[ac]anthracene^c^	DBacA	278
Dibenzo[ah]anthracene^a^	DBahA	278
Benzo[b]chrysene^c^	BbC	278
Picene^c^	Pic	278
Benzo[ghi]perylene-d_12_^b^	BghiP-d_12_	288
Benzo[ghi]perylene^a^	BghiP	278
Anthanthrene^c^	Anthan	276
Coronene^c^	Cor	300

^a^16 EPA PAHs; ^b^deuterated PAHs; ^c^nonpriority PAHs.

**Table 2:** Timed events and oven program used in direct injector mode.

Timed event			Oven program			
Event	Flow (mL·min^−1^)	Time (min)	Ramp	Rate (°C·min^−1^)	Temperature (°C)	Hold (min)
Split	0	−0.51	Initial	0	45	1
Split	50	1	1	20	200	0
Split	20	5	2	4	320	5

**Table 3:** Optimized conditions for thermal desorption system.

Primary desorption		Secondary desorption	
Parameter		Parameter	
Tube temperature	320°C	High trap temperature	320°C
Time	10 min	Low trap temperature	−10°C
Desorption flow	150 mL·min^−1^	Time	6 min
Inlet split flow	23 mL·min^−1^	Outlet split flow	10 mL·min^−1^

**Table 4:** TD-GC/MS method validation parameters for the 16 EPA PAHs in NIST SRM 1649b urban dust (*n*=10).

PAH	Experimental mean (ng)^a^	NIST-certified value (ng)^a^	RSD (%)	Recovery (%)	Accuracy^b^ (%)
Naph	3694 ± 1082	26.8 ± 3.0	46.3	13809	13709
Acy	6.97 ± 0.61	1.99 ± 0.24	13.9	351	251
Ace	1.57 ± 0.19	2.03 ± 0.41	19.5	77.3	−22.8
FL	2.06 ± 0.21	2.29 ± 0.67	16.4	89.9	−10.1
Phe	42.7 ± 3.3	45.3 ± 0.2	12.0	94.3	−5.70
Ant	12.6 ± 0.8	10.1 ± 0.2	9.82	125	25.1
Ft	59.6 ± 3.2	67.9 ± 0.4	8.56	87.7	−12.3
Pyr	51.8 ± 2.8	51.2 ± 1.4	8.56	101	1.01
BaA	19.3 ± 1.1	21.7 ± 0.5	8.70	88.7	−11.3
Chry	26.2 ± 1.3	31.3 ± 0.3	7.95	83.5	−16.5
BbFt + BjFt	75.0 ± 6.2	81.3 ± 2.3	13.0	92.2	−7.79
BkFt	16.1 ± 1.4	17.5 ± 0.5	13.4	91.9	−8.15
BaP	20.4 ± 1.5	25.4 ± 1.2	11.5	80.2	−19.8
IP	35.5 ± 1.9	29.7 ± 1.7	8.63	119	19.3
DBahA + DBacA	6.35 ± 0.93	6.02 ± 0.11	23.1	105	5.42
BghiP	37.2 ± 2.2	40.8 ± 0.4	9.31	91.1	−8.90
Average^c^	—	—	12.2	96.7	|12.4|

^a^Expanded uncertainty about the mean, with coverage factor, *k* = 2; ^b^accuracy = (experimental value − certified value) × 100/certified value; ^c^except Naph and Acy.

**Table 5:** Soxhlet extraction-GC/MS method validation parameters for the 16 EPA PAHs in SRM 1649b (*n*=7).

PAH	Experimental mean (ng)^a^	NIST-certified value (ng)^a^	RSD (%)	Recovery (%)	Accuracy^b^ (%)
Naph	67.3 ± 18.6	391 ± 35	33.8	17.2	−82.8
Acy	20.2 ± 5.9	79.9 ± 9.5	35.9	25.2	−74.8
Ace	25.0 ± 5.4	81.6 ± 16.5	26.5	30.7	−69.4
FL	32.6 ± 9.2	92.3 ± 14.4	34.5	35.3	−64.7
Phe	1215 ± 331	1668 ± 24	33.4	72.8	−27.2
Ant	104 ± 25	169 ± 1	32.5	61.8	−38.2
Ft	2392 ± 559	2573 ± 32	31.0	93.0	−7.05
Pyr	1914 ± 398	2054 ± 57	27.6	93.2	−6.79
BaA	808 ± 148	870 ± 20	24.3	92.9	−7.13
Chry	1632 ± 464	1256 ± 11	37.6	129	29.9
BbFt + BjFt	3144 ± 800	3260 ± 91	33.7	96.4	−3.58
BkFt	921 ± 319	702 ± 20	45.9	131	31.3
BaP	1019 ± 271	1018 ± 98	35.2	100	0.07
IP	1109 ± 296	1192 ± 65	35.4	93.1	−6.89
DBahA + DBacA	507 ± 176	241 ± 4	45.9	363	263
BghiP	2143 ± 580	1777 ± 32	35.8	120	20.6
Average^c^	—	—	34.9	120	|36.8|

^a^Expanded uncertainty about the mean, with coverage factor, *k* = 2; ^b^accuracy = (experimental value − certified value) × 100/certified value; ^c^except Naph, Acy, Ace, and FL.

**Table 6:** Reference PAH, RRFs, and the relative standard deviations (RSDs) for each nonpriority PAH.

Nonpriority PAH	Reference PAH	RRF	RSD (%)
BghiFt	BaA	1.29	6.68
BcP	BaA	0.76	13.2
CPP	BaA	0.31	15.1
Triph	BaA	0.69	16.1
Ret	BaA	1.13	25.6
BaFt	BkFt	0.99	11.3
BeP	BaP	1.31	4.10
Per	BaP	0.94	4.05
DBajA	IP	4.74	4.49
BbC	IP	1.08	6.16
Pic	IP	0.64	10.2
Anthan	BghiP	0.40	8.54
Cor	BghiP	0.54	15.8

**Table 7:** Descriptive statistics of the individual particle-bound PAH concentrations measured in the city of Bilbao.

PAH	N	Average (ng·m^−3^)	SD (ng·m^−3^)	Min. (ng·m^−3^)	Max. (ng·m^−3^)	5th percentile (ng·m^−3^)	95th percentile (ng·m^−3^)
Ace^∗^	180	0.22	0.27	4.00 × 10^−3^	1.69	0.02	0.85
FL^∗^	182	0.08	0.07	0.01	0.61	0.02	0.22
Phe^∗^	182	0.17	0.12	0.03	0.80	0.05	0.41
Ant^∗^	182	0.04	0.05	4.00 × 10^−3^	0.48	0.01	0.14
Ft^∗^	182	0.26	0.22	0.03	1.38	0.06	0.73
Pyr^∗^	182	0.27	0.23	0.02	1.48	0.05	0.73
BghiFt	118	0.20	0.19	0.01	0.87	0.03	0.60
BcP	181	0.06	0.06	4.00 × 10^−3^	0.40	8.00 × 10^−3^	0.20
CPP	73	0.07	0.16	3.00 × 10^−3^	0.95	5.00 × 10^−3^	0.48
BaA^∗^	182	0.16	0.22	0.01	1.45	0.02	0.62
Triph	164	0.14	0.13	0.02	0.74	0.03	0.43
Chry^∗^	182	0.22	0.24	0.03	1.35	0.04	0.81
BbFt^∗^ + BjFt	175	0.50	0.76	0.03	5.98	0.06	2.08
BkFt^∗^	174	0.18	0.23	0.01	1.41	0.03	0.64
BaFt	141	0.05	0.07	3.00 × 10^−3^	0.39	4.00 × 10^−3^	0.21
BeP	169	0.26	0.32	0.01	1.83	0.03	0.93
BaP^∗^	170	0.16	0.20	0.01	1.16	0.02	0.70
Per	159	0.03	0.04	2.00 × 10^−3^	0.19	4.00 × 10^−3^	0.14
DBajA	111	0.01	0.01	2.00 × 10^−4^	0.06	4.00 × 10^−4^	0.03
IP^∗^	161	0.17	0.24	1.00 × 10^−3^	1.52	0.01	0.70
DBahA^∗^ + DBacA	143	0.05	0.06	3.00 × 10^−4^	0.38	3.00 × 10^−3^	0.19
BbC	62	0.01	0.01	2.00 × 10^−3^	0.05	3.00 × 10^−3^	0.04
Pic	63	0.03	0.04	3.00 × 10^−3^	0.24	4.00 × 10^−3^	0.13
BghiP^∗^	174	0.20	0.19	0.01	1.03	0.04	0.66
Anthan	48	0.03	0.05	3.00 × 10^−3^	0.26	3.00 × 10^−3^	0.15
Cor	53	0.10	0.12	0.01	0.44	0.02	0.36

^∗^PAH listed as priority pollutant by US-EPA.

## Supplementary Materials

Supplementary Materials The representative SCAN chromatograms of the 16 EPAs and deuterated PAHs and also the SIM chromatograms of the target PAHs (16 EPA PAH + 15 PAH) obtained in the SRM 1649b analysis are shown in the supplementary material. Figure S1: chromatogram of 16 EPA and deuterated PAHs in SCAN mode, from 0 to 15 min: (1) Naph-d8, (2) Naph, (3) Bph-d10, (4) Acy, (5) Ace, and (6) FL. Figure S2: chromatogram of 16 EPA and deuterated PAHs in SCAN mode, from 15 to 20.5 min: (7) Phe-d10, (8) Phe, (9) Ant, (10) Ft, (11) Pyr-d10, and (12) Pyr. Figure S3: chromatogram of 16 EPA and deuterated PAHs in SCAN mode, from 22.5 to 35.5 min: (13) BaA-d12, (14) BaA, (15) Chry, (16) BbFt, (17) BkFt, (18) BaP-d10, (19) BaP, (20) IP, (21) DBahA, (22) BghiP-d12, and (23) BghiP. Figure S4: PAHs and deuterated PAHs in SIM windows (m/z 226, 240, 228, and 234) in the analysis of NIST SRM 1649b dust. Figure S5: PAHs and deuterated PAHs in SIM windows (m/z 252 and 264) in the analysis of NIST SRM 1649b dust. Figure S6: PAHs and deuterated PAHs in SIM windows (m/z 276, 288, and 278) in the analysis of NIST SRM 1649b dust. Figure S7: PAHs and deuterated PAHs in m/z 300 SIM window in the analysis of NIST SRM 1649b dust.
