# Supplementary material for: Optimization and Validation of Thermal Desorption Gas Chromatography-Mass Spectrometry for the Determination of Polycyclic Aromatic Hydrocarbons in Ambient Air
Source: J Anal Methods Chem. 2018 May 2;2018:8734013. doi: 10.1155/2018/8734013 (PMC5954884; doi:10.1155/2018/8734013)
Supplement: Supplementary Materials — The representative SCAN chromatograms of the 16 EPAs and deuterated PAHs and also the SIM chromatograms of the target PAHs (16 EPA PAH + 15 PAH) obtained in the SRM 1649b analysis are shown in the supplementary material. Figure S1: chromatogram of 16 EPA and deuterated PAHs in SCAN mode, from 0 to 15 min: (1) Naph-d8, (2) Naph, (3) Bph-d10, (4) Acy, (5) Ace, and (6) FL. Figure S2: chromatogram of 16 EPA and deuterated PAHs in SCAN mode, from 15 to 20.5 min: (7) Phe-d10, (8) Phe, (9) Ant, (10) Ft, (11) Pyr-d10, and (12) Pyr. Figure S3: chromatogram of 16 EPA and deuterated PAHs in SCAN mode, from 22.5 to 35.5 min: (13) BaA-d12, (14) BaA, (15) Chry, (16) BbFt, (17) BkFt, (18) BaP-d10, (19) BaP, (20) IP, (21) DBahA, (22) BghiP-d12, and (23) BghiP. Figure S4: PAHs and deuterated PAHs in SIM windows (m/z 226, 240, 228, and 234) in the analysis of NIST SRM 1649b dust. Figure S5: PAHs and deuterated PAHs in SIM windows (m/z 252 and 264) in the analysis of NIST SRM 1649b dust. Figure S6: PAHs and deuterated PAHs in SIM windows (m/z 276, 288, and 278) in the analysis of NIST SRM 1649b dust. Figure S7: PAHs and deuterated PAHs in m/z 300 SIM window in the analysis of NIST SRM 1649b dust. [file 8734013.f1.pdf]

Supplementary information

Figure S1. Chromatogram of 16 EPA and deuterated PAHs in SCAN mode, from 0 to 15 min: 1) Naph-d_8, 2) Naph, 3) Bph-d_{10}, 4) Acy, 5) Ace, 6) FL.

Figure S2. Chromatogram of 16 EPA and deuterated PAHs in SCAN mode, from 15 to 20.5 min: 7) Phe-d_{10}, 8) Phe, 9) Ant, 10) Ft, 11) Pyr-d_{10}, 12) Pyr.

Figure S3. Chromatogram of 16 EPA and deuterated PAHs in SCAN mode, from 22.5 to 35.5 min: 13) BaA-d_{12}, 14) BaA, 15) Chry, 16) BbFt, 17) BkFt, 18) BaP-d_{10}, 19) BaP, 20) IP, 21) DBahA, 22) BghiP-d_{12}, 23) BghiP.

Figure S4. PAHs and deuterated PAHs in SIM windows (m/z 226,240,228 and 234) in the analysis of NIST SRM 1649b dust.

Figure S5. PAHs and deuterated PAHs in SIM windows (m/z 252 and 264) in the analysis of NIST SRM 1649b dust.

Figure S6. PAHs and deuterated PAHs in SIM windows (m/z 276, 288 and 278) in the analysis of NIST SRM 1649b dust.

Figure S7. PAHs and deuterated PAHs in m/z 300 SIM window in the analysis of NIST SRM 1649b dust.

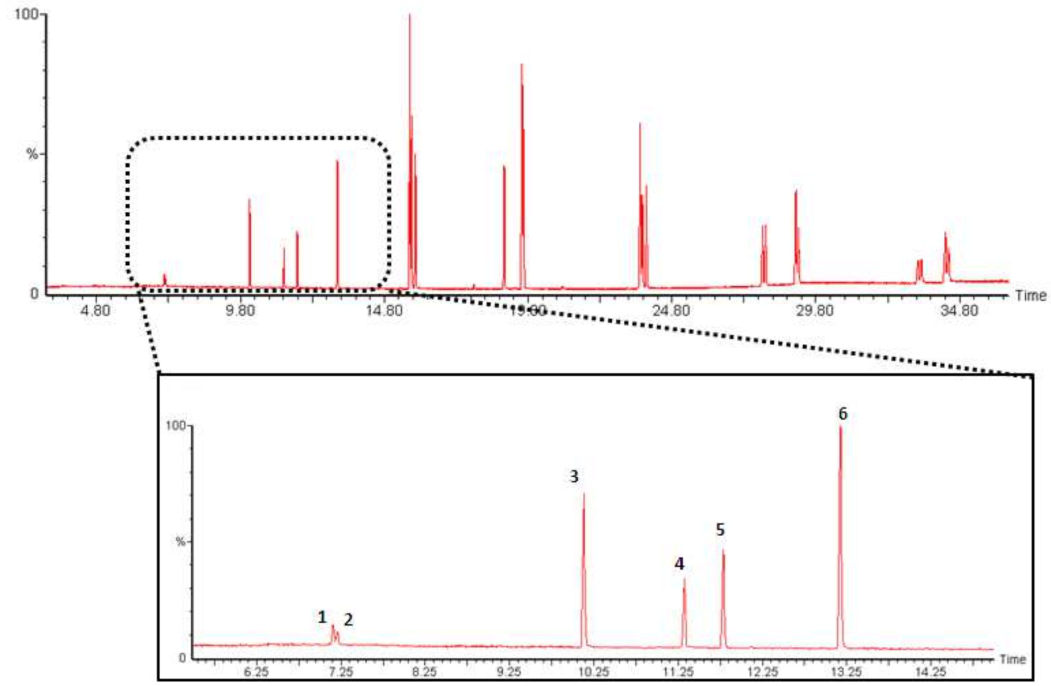

Figure S1. Chromatogram of 16 EPA and deuterated PAHs in SCAN mode, from 0 to 15 min: 1) Naph-d_8, 2) Naph, 3) Bph-d_{10}, 4) Acy, 5) Ace, 6) FL.

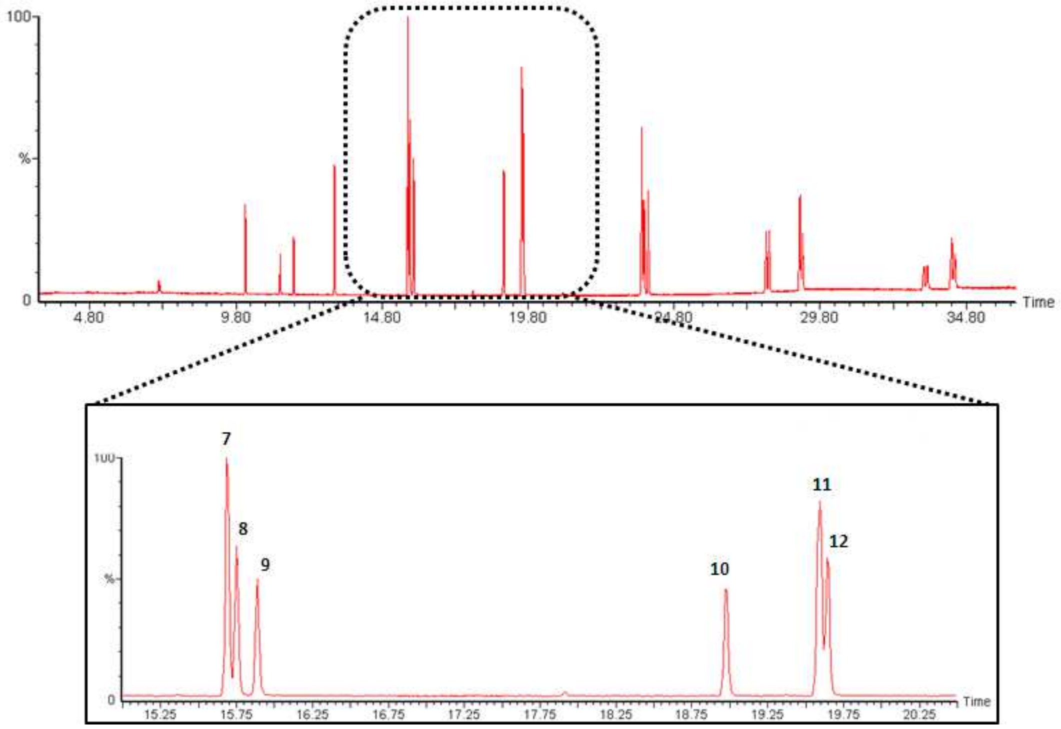

Figure S2. Chromatogram of 16 EPA and deuterated PAHs in SCAN mode, from 15 to 20.5 min: 7) Phe-d_{10}, 8) Phe, 9) Ant, 10) Ft, 11) Pyr-d_{10}, 12) Pyr.

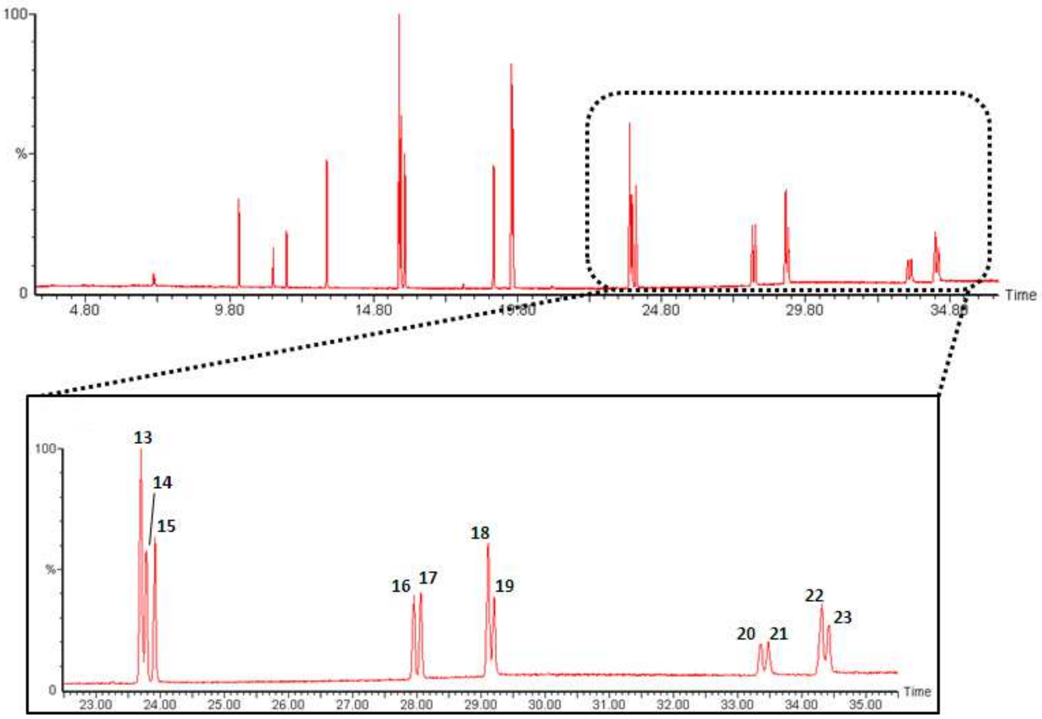

Figure S3. Chromatogram of 16 EPA and deuterated PAHs in SCAN mode, from 22.5 to 35.5 min: 13) BaA-d_{12}, 14) BaA, 15) Chry, 16) BbFt, 17) BkFt, 18) BaP-d_{10}, 19) BaP, 20) IP, 21) DBahA, 22) BghiP-d_{12}, 23) BghiP.

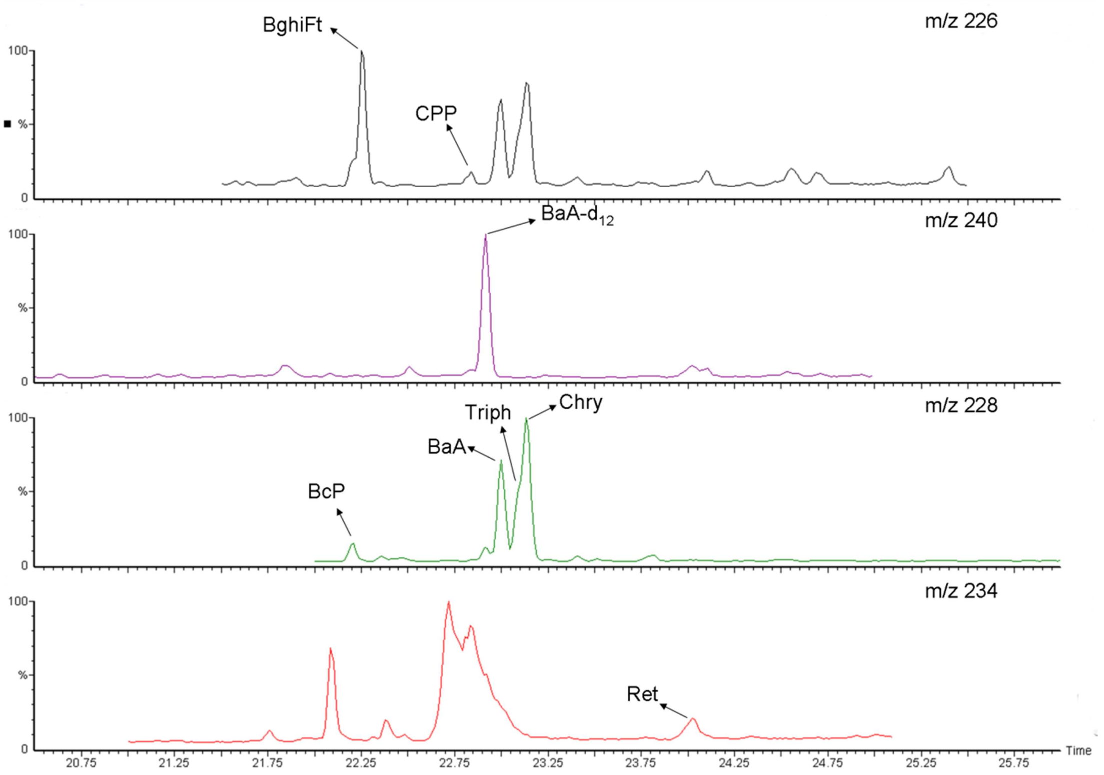

Figure S4. PAHs and deuterated PAHs in SIM windows (m/z 226,240,228 and 234) in the analysis of NIST SRM 1649b dust.

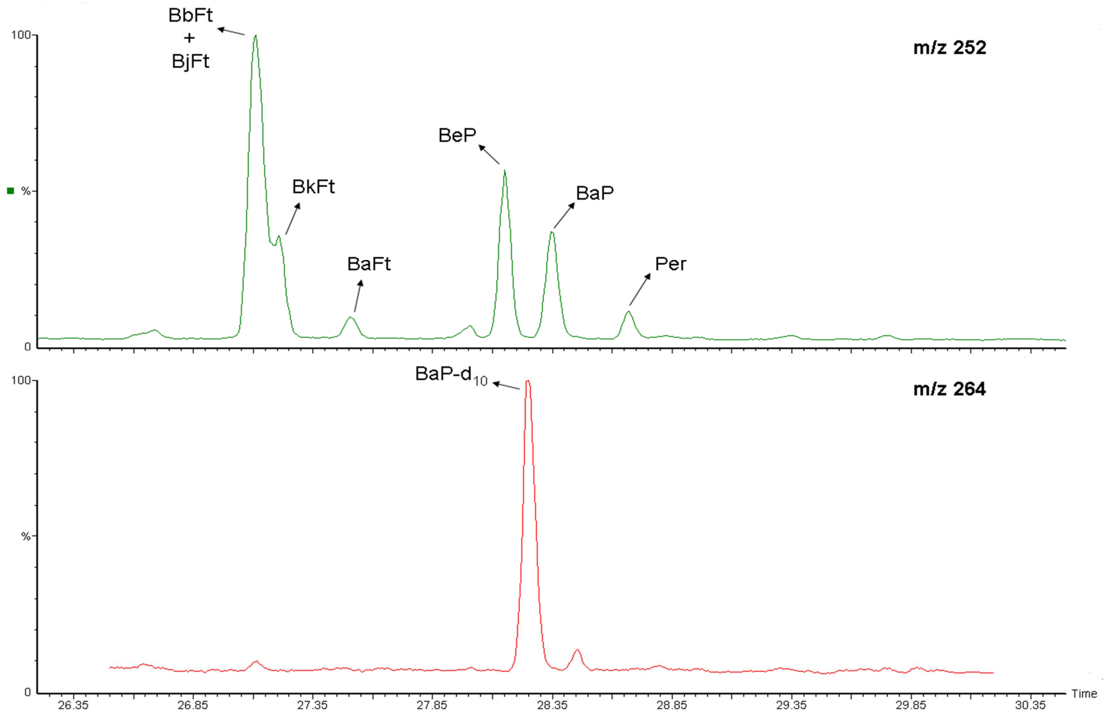

Figure S5. PAHs and deuterated PAHs in SIM windows (m/z 252 and 264) in the analysis of NIST SRM 1649b dust.

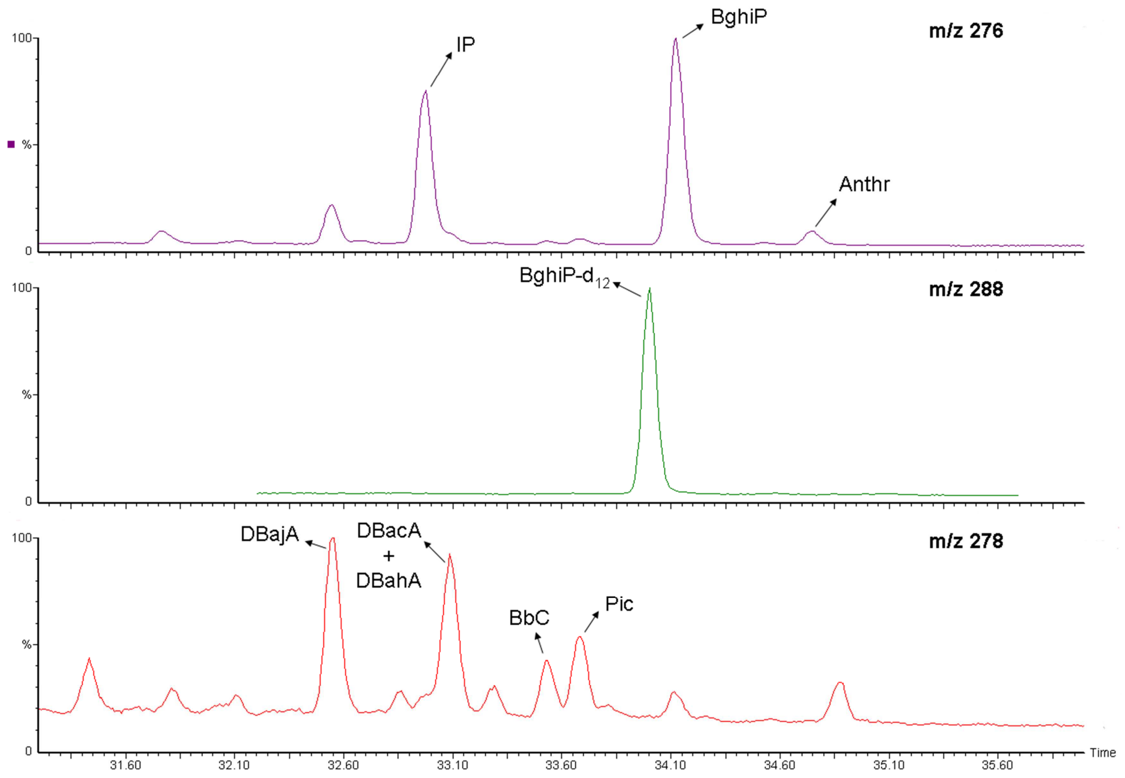

Figure S6. PAHs and deuterated PAHs in SIM windows (m/z 276, 288 and 278) in the analysis of NIST SRM 1649b dust.

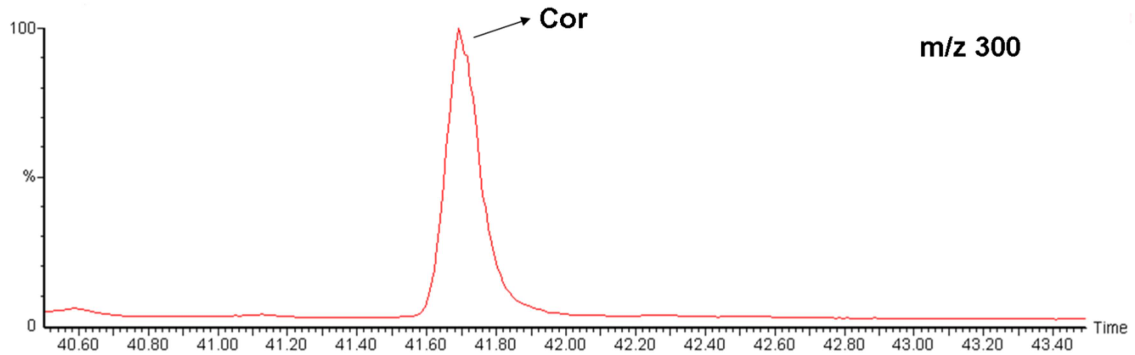

Figure S7. PAHs and deuterated PAHs in m/z 300 SIM window in the analysis of NIST SRM 1649b dust.
